# Comparative analyses of long non-coding RNA in lean and obese pigs

**DOI:** 10.18632/oncotarget.18269

**Published:** 2017-05-26

**Authors:** Lin Yu, Lina Tai, Lifang Zhang, Yi Chu, Yixing Li, Lei Zhou

**Affiliations:** ^1^ State Key Laboratory for Conservation and Utilization of Subtropical Agro-Bioresources, College of Animal Science and Technology, Guangxi University, Nanning, P.R. China

**Keywords:** lncRNA, pig, obesity, QTL

## Abstract

**Objectives:**

Current studies have revealed that long non-coding RNA plays a crucial role in fat metabolism. However, the difference of lncRNA between lean (Duroc) and obese (Luchuan) pig remain undefined. Here, we investigated the expressional profile of lncRNA in these two pigs and discussed the relationship between lncRNA and fat deposition.

**Materials and Methods:**

The Chinese Luchuan pig has a dramatic differences in backfat thickness as compared with Duroc pig. In this study, 4868 lncRNA transcripts (including 3235 novel transcripts) were identified. We determined that patterns of differently expressed lncRNAs and mRNAs are strongly tissue-specific. The differentially expressed lncRNAs in adipose tissue have 794 potential target genes, which are involved in adipocytokine signaling pathways, the PI3k-Akt signaling pathway, and calcium signaling pathways. In addition, differentially expressed lncRNAs were located to 13 adipose-related quantitative trait loci which include 65 QTL_ID. Subsequently, lncRNA and mRNA in the same QTL_ID were analyzed and their co-expression in two QTL_ID were confirmed by qPCR.

**Conclusions:**

Our study provides an insight into mechanism behind the fat metabolic differences between the two breeds and lays an important groundwork for further research regarding the regulatory role of lncRNA in obesity development.

## INTRODUCTION

Obesity has become a major health concern around the world and is the main risk factor for non-alcoholic fatty liver disease (NAFLD), type 2 diabetes and cardiovascular diseases (CVDs) [1]. Therefore, the study of fat deposition and its mechanism is of great benefit for prevention and treatment of obesity and its related diseases. The Luchuan pig is a typical obese breed as it has higher intramuscular fat and backfat thickness compared with the Duroc breed. They are good models to investigate the regulatory mechsniam of fat metabolism.

Long non-coding RNAs (lncRNAs) are defined as non-coding RNAs of at least 200 nucleotides. In the past, lncRNAs were considered to be “evolutionary junk” or transcriptional “noise” along with other non-coding RNAs [2, 3]. However, in recent years, as the rapid development of technologies has facilitated analysis of the “transcriptome”, there is increasing evidence that lncRNAs play a crucial role in many biological processes [4, 5], such as telomere homeostasis and chromosome replication [6–8], control of nuclear architecture and translation [9], X-chromosome inactivation [10], regulation of epigenetic modifications [11], control of mRNA and protein stability [12, 13], and regulation of miRNA activity [14, 15]. As research into lncRNA increased, many databases were established and included lncRNA data for both domesticated animals and poultry. At the time of publication, a total of 12,103 pig lncRNAs, 8,923 chicken lncRNAs and 8,250 cow lncRNAs are included in the ALDB database [16]. While analyzing lncRNA expression in pig, Zhao et al. developed a systematic protocol for the identification and characterization of lncRNAs in fetal porcine skeletal muscle [17]. Moreover, an antisense lncRNA of the *PU.1* gene was identified, which can form a sense-antisense RNA duplex to promote adipogenesis [18]. Wang et al. investigated the lncRNAs in porcine endometrial tissue samples using RNA-seq [19]. Currently, porcine fat deposition is less well understood. Toward that end, in order to compare the lncRNA expression differences between the lean and obese breeds, lncRNA sequences were obtained from three different types of tissues (liver, muscle and fat) of Luchuan and Duroc pigs.

In this study, we identified differentially expressed lncRNA molecules and predicted their target genes. Moreover, the correlation between the identified lncRNA molecules and QTL were investigated. These results will provide a useful resource to further explore the role of lncRNAs in fat deposition.

## RESULTS

### Overview of lncRNA sequencing data

**Figure 1:**
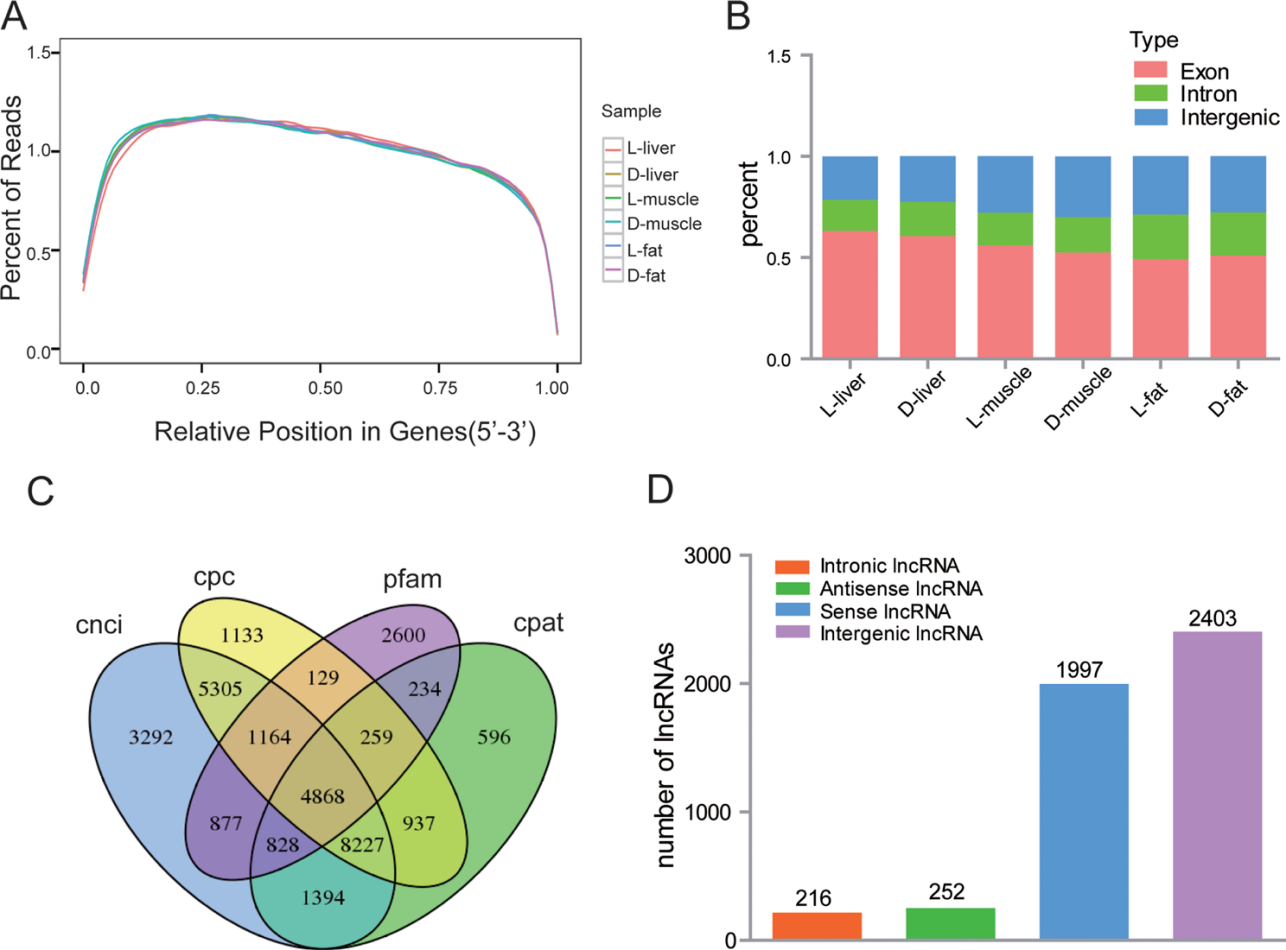
Overview of lncRNA sequencing data (**A**) The distribution of mapped reads on mRNA (5′–3′). Data in reflects the percentage of mapped reads assigned to all regions of mRNA. The location of the normalized mRNA is on the horizontal (x) axis; the percentage of reads as compared to total mapped reads for the position is on the vertical (y) axis. As the reference mRNA is different in length, each mRNA is divided into 100 intervals by length. (**B**) Reads mapped to different regions of the genome. (**C**) Venn diagrams show the result of four computational approaches. 4868 candidate lncRNAs were identified from a intersection results from the CNCI (coding-non-coding index), CPC (coding potential calculator), Pfam (protein folding domain database), and CPAT (coding potential assessing tool). (**D**) The type and number of predicted long non-coding RNAs (lncRNAs). Intronic lncRNA: lncRNA transcript from the intron region of gene; Antisense lncRNA: lncRNA has opposite transcriptional direction compared to adjacent mRNA; Sense lncRNA: lncRNA has the same transcriptional direction as the adjacent mRNA; Intergenic lncRNA: lncRNA transcribed from a position between two genes.

After 180 days of identical feeding conditions, the average backfat thickness of Luchuan pigs was 35.33 ± 0.57 mm while that of the Duroc pigs was 12 ± 1.00 mm. Next, three tissue samples (liver, muscle and fat) were collected from three animals from each porcine breed: L-liver (Luchuan liver), D-liver (Duroc liver), L-muscle (Luchuan muscle), D-muscle (Duroc muscle), L-fat (Luchuan fat) and D-fat (Duroc fat). Total RNA from each sample was sequenced by using the Illumina HiSeq 2500 platform. A total of 16.84G, 14.82G, 13.37G, 12.26G, 15.56G and 14.00G clean data was generated in the L-liver, D-liver, L-muscle, D-muscle, L-fat and D-fat samples, respectively. The GC content averaged between 51.84 and 56.77% while the Q30 ranged between 95.08 and 96.16%. These results show that the quality of our six libraries was good and suitable for subsequent analysis. Next, the clean reads were aligned to the reference genome (Susscrofa 10.2) using Tophat v2.1.0 (Table 1). More than 68.54% of clean reads were uniq mapped in each sample. An average of 40.68% of clean reads were mapped to sense strand and 39.43% of clean reads were mapped to antisense strand. A high quality library is a necessary for lncRNA sequencing; therefore, the data from each read's relative position in gene (5′–3′) were analyzed to ensure the quality of these six samples. Obviously, the vast majority of reads were evenly distributed throughout the gene by random sampling, which indicates that the quality and homogeneity of the samples was good (Figure 1A). Moreover, the ratio of reads corresponding to exon, intron, and intergenic regions was different (Figure 1B), suggesting that the RNA expression profiles were tissue specific.

**Table 1:** Categorization of reads and basic characteristics of lncRNAs in Luchuan and Duroc pigs

	Luchuan			Duroc			
Sample ID	L-liver	L-muscle	L-fat	D-liver	D-muscle	D-fat	Average
Clean Data	16840128832	13368920108	15559684336	14822619834	12261255102	14001588618	14475699472
GC (%)	51.84	55.57	55.9	54.32	56.77	56.18	55.09
N (%)	0.01	0.01	0.01	0.01	0.01	0.01	0.01
Q30 (%)	95.79	95.71	95.46	96.16	95.68	95.08	95.65
Clean Reads	113662050	91521480	106758414	102156440	84311820	96436194	99141066
Mapped Reads	93369950 (82.15%)	70725019 (77.28%)	82473446 (77.25%)	86378716 (84.56%)	65642547 (77.86%)	78681690 (81.59%)	79545228 (80.12%)
Uniq Mapped Reads	71734450 (76.83%)	51258518 (72.48%)	58949568 (71.48%)	61811541 (71.56%)	44992237 (68.54%)	56815782 (72.21%)	57593683 (72.18%)
Multiple Mapped Reads	21635500 (23.17%)	19466501 (27.52%)	23523878 (28.52%)	24567175 (28.44%)	20650310 (31.46%)	21865908 (27.79%)	21951545 (27.82%)
Reads Map to ‘+’	47306223 (41.62%)	35819308 (39.14%)	42098432 (39.43%)	43643606 (42.72%)	33366249 (39.57%)	40109194 (41.59%)	40390502 (40.68%)
Reads Map to ‘−’	46063727 (40.53%)	34905711 (38.14%)	40375014 (37.82%)	42735110 (41.83%)	32276298 (38.28%)	38572496 (40.00%)	39154726 (39.43%)

Triplicate tissue samples were collected from three pigs of each species. GC (%) is the percentage of G and C bases / total nucleotides; *N* (%) is the percentage of unrecognized bases / total nucleotides; Q30 (%) is the percentage of bases’ mass greater than or equal Q30 in the clean data; Uniq Mapped Reads is the number and percentage of reads that mapped to a unique position in the reference genome in the clean reads; Multiple Mapped Reads is the number and percentage of reads that mapped to multiple positions in the reference genome in the clean reads; Reads Map to ‘+’ is the number of clean reads mapped to the sense strand; Reads Map to ‘−’ is the number of clean reads mapped to antisense strand.

### Identification of lncRNAs expressed in liver, muscle, and fat tissue of Luchuan and Duroc pig breeds

All mapped reads of the six libraries were assembled using Cufflinks [20]; then, the assembled transcripts were filtered using a rigorous method. Transcripts which were >200 bp in length, contained two exons, had at least three reads coverage, and had a 0.1 FPKM value were retained. As lncRNA do not encode proteins, the protein coding potential of the remaining transcripts was determined using four separate protocols: CPC, CNCI, CPAT and pafm. Finally, 4868 lncRNA transcripts were identified (Figure 1C), including 2403 lincRNAs (49.36%), 252 anti-sense lncRNAs (5.18%), 216 intronic lncRNAs (4.44%), and 1997 sense lncRNAs (41.02%) (Figure 1D). Moreover, 3235 novel lncRNA transcripts were revealed by blasting their sequences in NONCODE and lncRNAdb.

**Figure 2:**
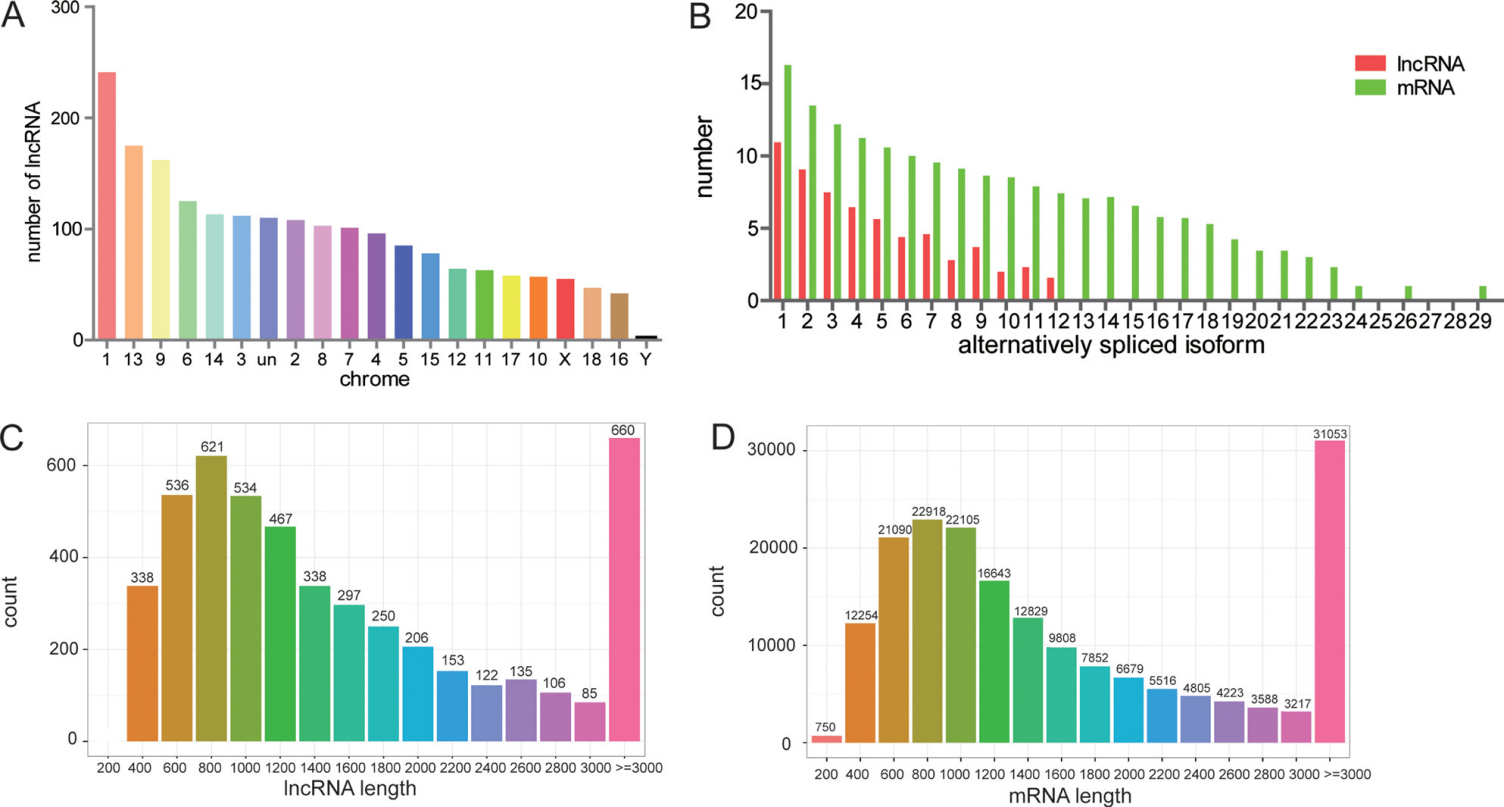
Features of lncRNAs and mRNAs in the genome (**A**) LncRNAs distribution by chromosome. (**B**) Alternatively spliced isoforms per lncRNA and mRNA molecule. (**C&D**) Distribution of lncRNA and mRNA molecules by length.

**Figure 3:**
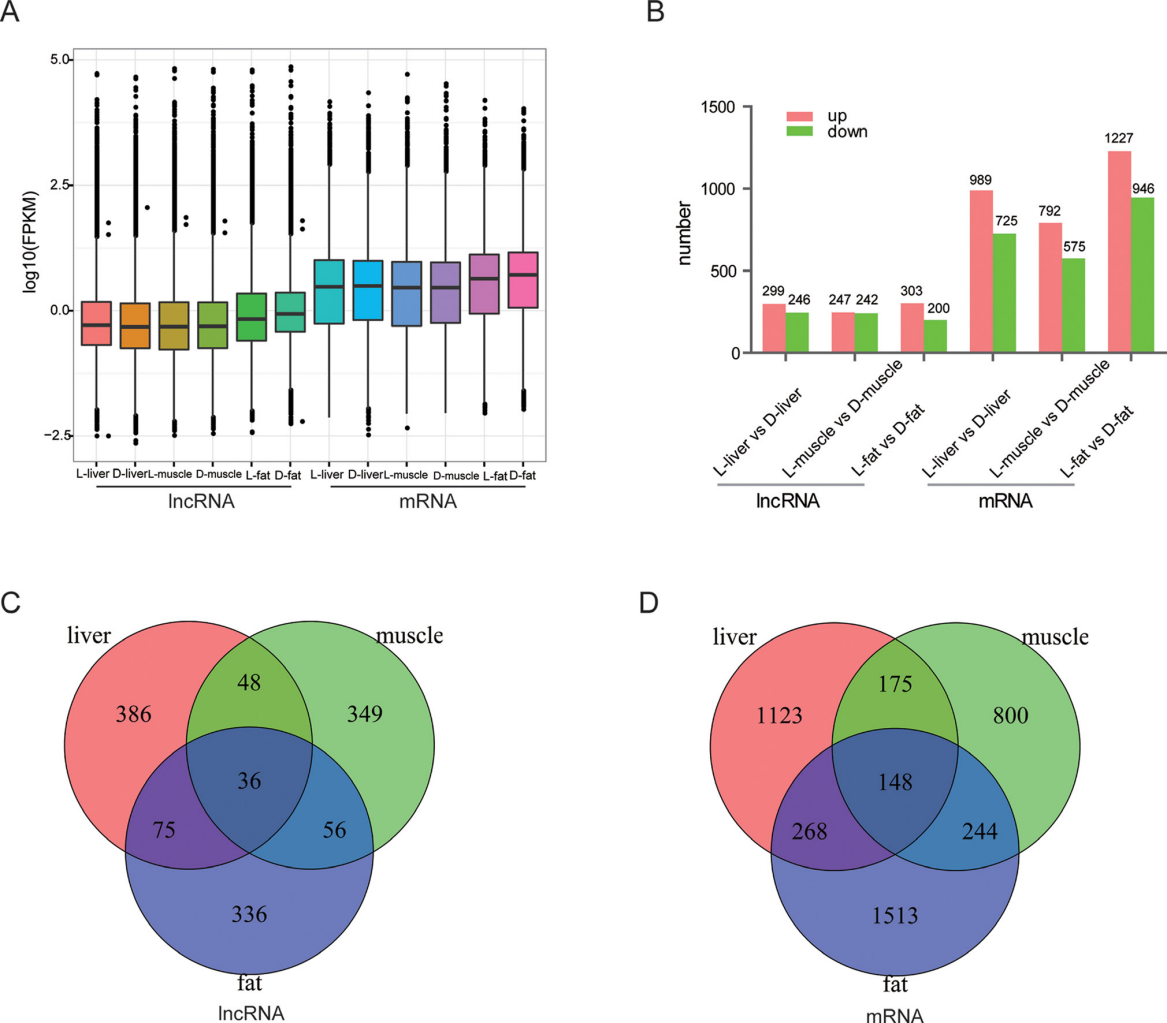
Differential expression of lncRNAs and mRNAs by tissue (**A**) The number of differentially expressed lncRNAs and mRNAs by tissue. Red bar represents up-regulated transcripts and the green bar represent down-regulate transcripts. (**B**) Expression profiles of lncRNA and mRNA in each tissue category. We used log10(FPKM) as the final data to indicate expression level. (**C**, **D**) Tissue-specific expression of lncRNAs and mRNA.

These 4868 lncRNA transcripts were distributed throughout all chromosomes found in pig, although chromosome 1 contained the greatest number of lncRNAs (Figure 2A). On the whole, there were fewer alternatively spliced isoforms per lncRNA molecule as compared with mRNA molecules (Figure 2B). Lengths between 600~1200 bp and ≥ 3000 bp from both lncRNA and mRNA molecules were most common (Figure 2C–2D). All expression information of lncRNAs and genes are shown in Supplementary Tables 1, 2. We noticed that all the lncRNAs tended to be expressed at a lower level than the protein-coding genes (Figure 3A). Then, all of differently expressed transcripts were filtered to include only those transcripts with a false discovery rate (FDR) < 0.05 and fold change ≥ 2 or ≤ 0.5; the FDR is obtained by from adjusting the *p-value*, and the fold change was obtain from gene expression of Luchuan / gene expression of Duroc pig. In adipose tissue, 503 lncRNA and 2173 mRNA molecules were detected (Figure 3B). All of the differentially expressed lncRNAs and mRNAs are shown in Supplementary Tables 3, 4. Two Venn diagrams depict the differentially expressed lncRNA and mRNA molecules in each of the three tissue types (Figure 3C–3D). A total of 386, 349 and 336 differentially expressed lncRNAs appear to be specific for the liver, muscle and fat, respectively. Tissue specific lncRNAs were a major proportion of the differentially expressed lncRNAs. Similarly, 1123, 800 and 1513 differently expressed mRNAs appear to be specific for the liver, muscle and fat, respectively.

**Figure 4:**
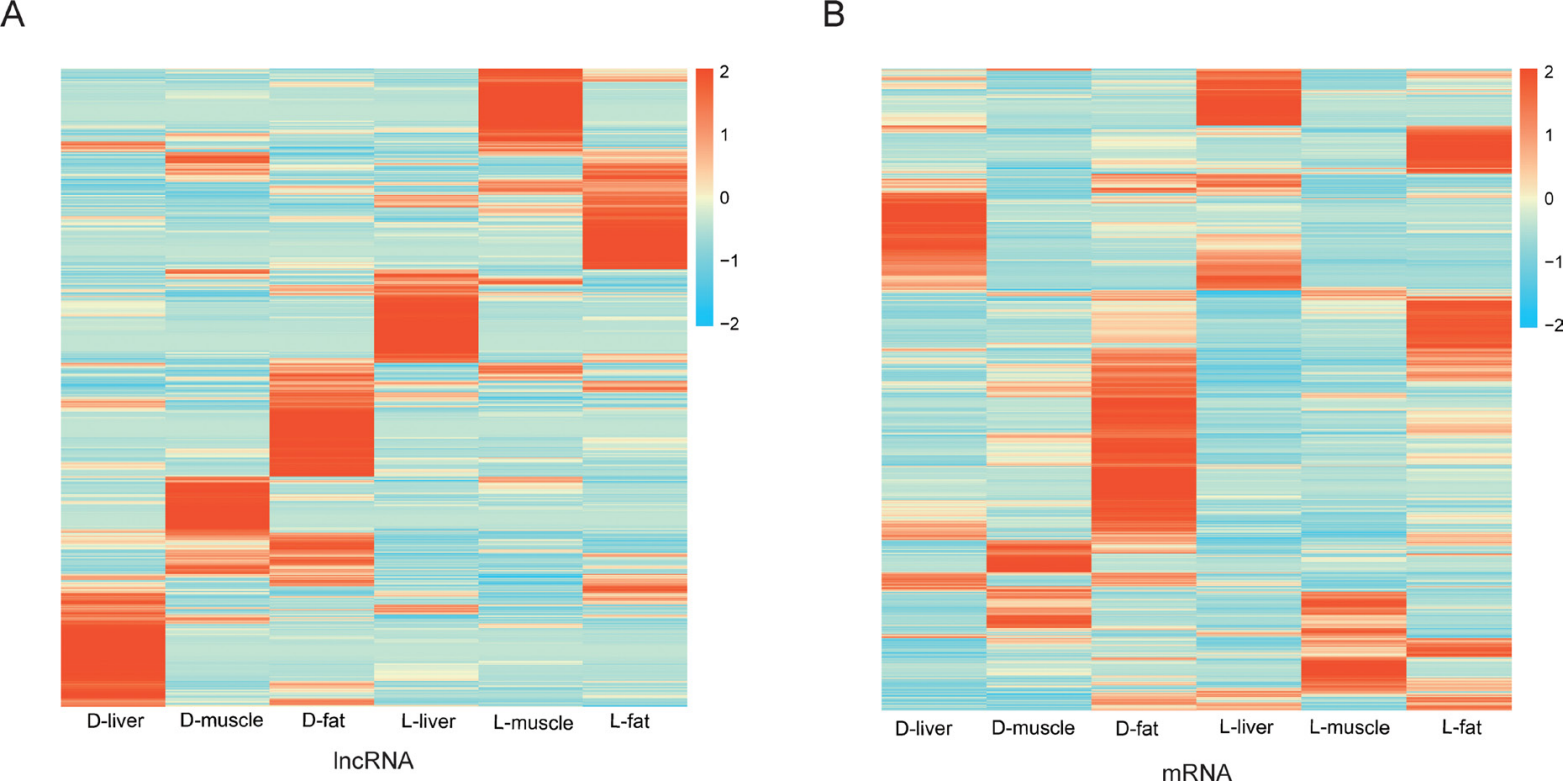
Heat-map of differently expression lncRNAs and mRNAs (**A**) Cluster heat-map of differentially expressed lncRNAs from each sample. (**B**) Cluster heat-map of differentially expressed mRNAs from each sample.

Next, 1286 and 4271 unique and differentially expressed lncRNAs and mRNA were used to perform a tissue-specific clustering analysis. As shown in the heat map, the up-regulated lncRNAs were divided into six clusters. The expression pattern of Duroc lncRNAs (D-liver, D-muscle and D-fat ) was distinct from the expression of Luchuan lncRNAs (L-liver, L-muscle and L-fat ) (Figure 4A). In contrast, according to the heat map of differentially expressed mRNA transcripts, the cluster of up-regulated mRNA molecules was similar between the Luchuan liver and Duroc liver samples as well as between the Luchuan muscle and Duroc muscle samples. However, the Luchuan fat and Duroc fat samples expressed distinct mRNA molecules (Figure 4B). In order to further understand the potential function of lncRNA in fat deposition, the target genes of the lncRNAs were predicted. A portion of some lncRNA molecules and their target gene are presented in Table 2. All of the target genes are listed in Supplementary Table 5.

**Table 2:** Differentially expressed lncRNAs and their target mRNA in each tissue

Tissue	lncRNA_ID	Fold change	Regulated	Gene_ID	Gene name	Fold change	Regulated
liver	TCONS_00109066	222.8439	up	ENSSSCG00000014368	–	25.75714	up
liver	TCONS_00156770	175.7342	up	ENSSSCG00000003967	ZMYND12	0.235277	down
liver	TCONS_00176571	79.80171	up	ENSSSCG00000002627	GSTA4	2.524484	up
liver	TCONS_00130158	0.007197	down	ENSSSCG00000006155	ZBTB10	0.277276	down
liver	TCONS_00062308	0.005966	down	ENSSSCG00000024537	–	5.06822	up
liver	TCONS_00143452	0.00467	down	ENSSSCG00000000878	DEPDC4	0.321732	down
muscle	TCONS_00013468	123.2883	up	ENSSSCG00000005087	SIX1	0.428436	down
muscle	TCONS_00108745	93.70243	up	ENSSSCG00000014083	ANKDD1B	2.762261	up
muscle	TCONS_00136478	81.86808	up	ENSSSCG00000006506	SYT11	0.325533	down
muscle	TCONS_00024736	0.006558	down	ENSSSCG00000023031	TNNT2	7.496831	up
muscle	TCONS_00018030	0.004239	down	ENSSSCG00000005087	SIX1	0.428436	down
muscle	TCONS_00008591	0.002903	down	ENSSSCG00000004602	TEX9	0.160484	down
fat	TCONS_00027769	167.275	up	ENSSSCG00000010837	FAM177B	63.01933	up
fat	TCONS_00181629	149.6883	up	ENSSSCG00000027340	–	112.1	up
fat	TCONS_00109934	73.74533	up	ENSSSCG00000013148	GLYATL2	59.994	up
fat	TCONS_00066375	0.01899	down	ENSSSCG00000010069	DERL3	18.56326	up
fat	TCONS_00194244	0.017954	down	ENSSSCG00000014861	MOGAT2	0.156778	down
fat	TCONS_00174306	0.007341	down	ENSSSCG00000001826	CCDC37	13.31454	up

The top three up regulated and down regulated lncRNAs with its target mRNA in each tissue.

### Gene ontology (GO) and KEGG pathway enrichment analysis

**Figure 5:**
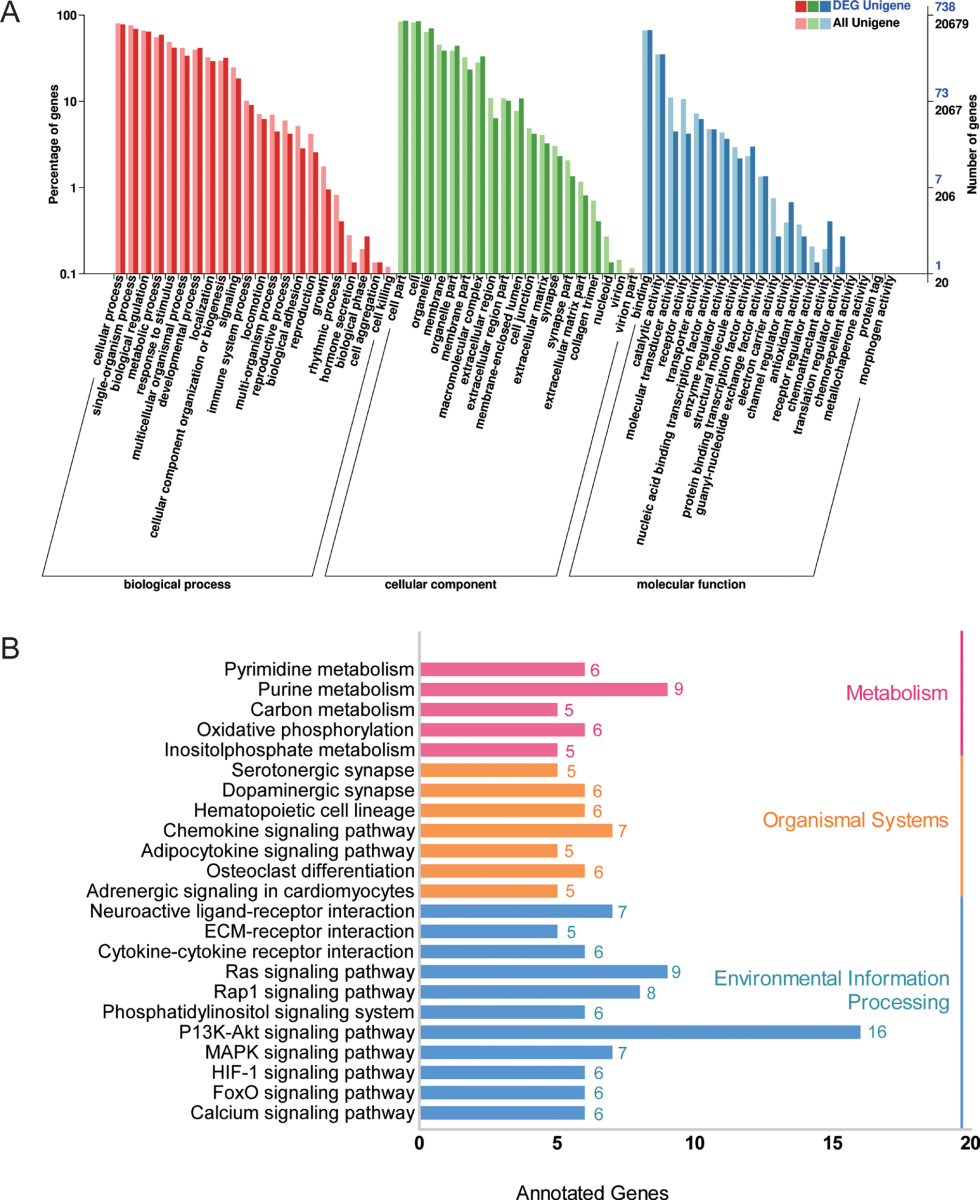
GO and KEGG analysis of target genes in adipose tissue (**A**) Gene Ontology analysis of target genes of differentially expressed lncRNAs from adipose tissue (L-fat vs D-fat). DEG Unigene: differentially expressed genes number in all annotation Biological Process GO term. All Unigene: Unigene number in all annotation Biological Process GO term. (**B**) KEGG pathway enrichment analysis of target genes of differentially expressed lncRNAs from adipose tissue (L-fat vs D-fat).

Considering the adipose tissue had the greatest number (2173) of differentially expressed mRNAs and is a major organ for fat deposition, a gene ontology (GO) analysis was performed for the adipose tissues. The most enriched GO terms are shown in Figure 5A; complete information is listed in Supplementary Table 6. We primarily focused on genes involved in electron carrier activity and antioxidant activity as these functions may be involved in the regulation of fat deposition. Meanwhile, these target genes also underwent a KEGG analysis to determine the potential biological function of the identified lncRNAs. The data showed that some pathways related to fat metabolism and energy metabolism were significantly enriched, such as signaling pathways associated with adipocytokines, calcium signaling, MAPK, FOXO and PI3k/Akt (Figure 5B and Supplementary Table 7). To our surprise, there were 16 target genes that function as part of the PI3k-Akt signaling pathway, which is closely related to insulin signaling pathway [21].

### QTL location analysis of DE-lncRNAs and quantitative validation

Due to the tight connection between QTL and traits, and to further explore the role of differentially expressed lncRNA molecules on fat deposition, a correlation analysis was performed between lncRNA and fat-associated QTL by mapping differentially expressed lncRNAs onto pig QTL regions. This analysis indicated that 275 differentially expressed lncRNAs are located in 13 fat-associated QTL (Supplementary Table 8). At the same time, differentially expressed mRNAs were mapped onto these 13 QTL and 498 mRNAs were found to localize to the 13 QTL as well. Genes associated with the trait “abdominal fat weight” had the greatest number of associated differentially expressed lncRNAs (138) and mRNAs (513). The trait, “average backfat thickness”, had the second highest number of associated differentially expressed lncRNAs (89) and mRNAs (306) (Table 3).

**Table 3:** Differentially expressed lncRNA and mRNA molecules (L-fat VS D-fat) assigned into QTL trait regions

Trait	Number of QTL_ID	All DE-lncRNAs	Up-lncRNA	Down-lncRNA	All DE-mRNAs	Up-mRNA	Down-mRNA
Abdominal fat percentage	2	4	3	1	8	5	3
Abdominal fat weight	17	138	79	59	513	308	205
Adipocyte diameter	6	23	13	10	97	53	44
Arachidic acid content	2	3	1	2	3	2	1
Arachidonic acid content	1	3	2	1	1	1	0
Average backfat thickness	28	89	60	29	306	167	139
backfat above muscle dorsi	2	5	2	3	82	53	29
backfat at last rib	1	2	0	2	17	5	12
Backfat at rump	1	1	0	1	8	4	4
Backfat between 6th and 7th ribs	1	2	0	2	8	5	3
Backfat weight	2	3	2	1	15	5	10
Loin fat percentage	1	1	1	0	0	0	0
Percentage of backfat and leaf fat in carcass	1	1	1	0	7	3	4

Up-lncRNA: up regulated lncRNA number; Down-lncRNA: down regulated lncRNA number; Up-mRNA: up regulated mRNA number; Down-mRNA: down regulated mRNA number.

**Figure 6:**
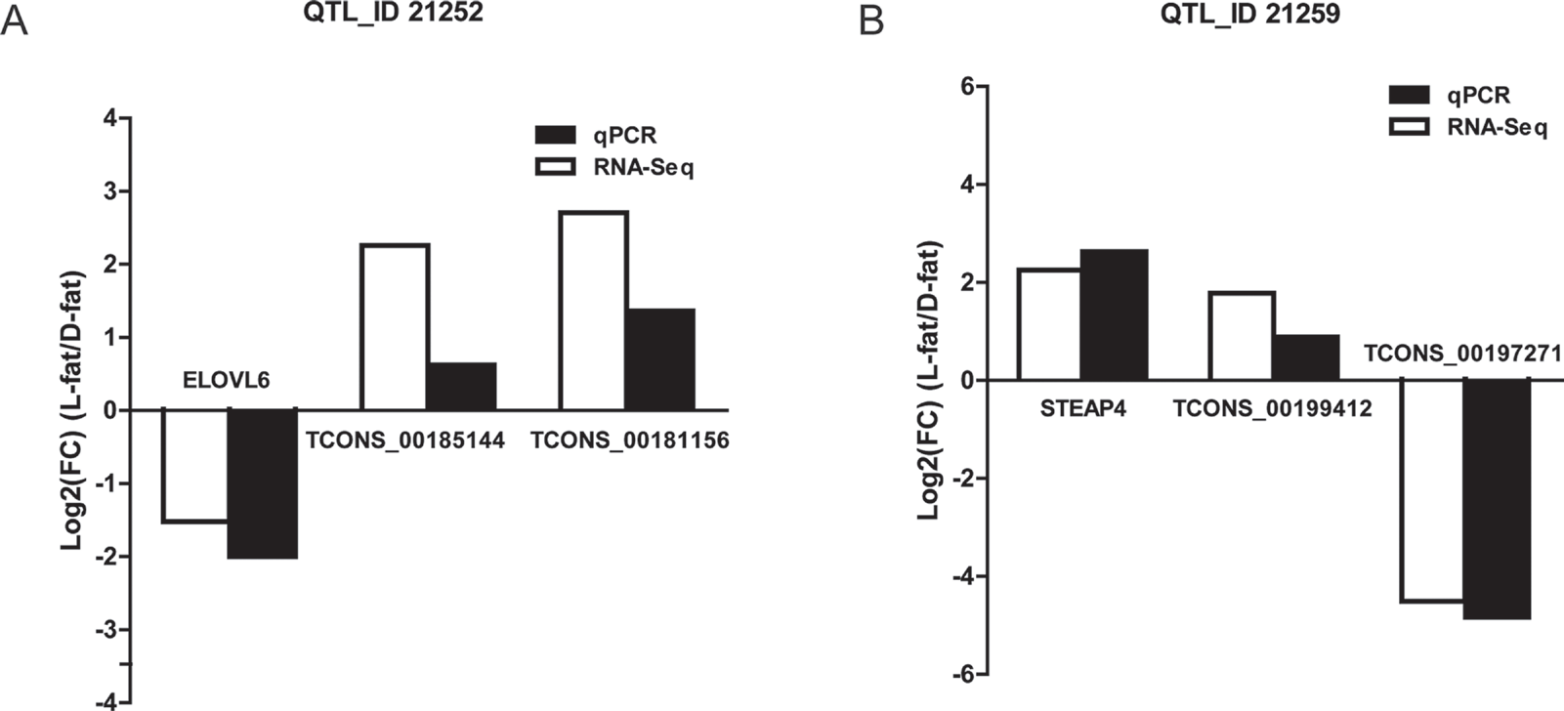
Co-expression of transcripts validation via quantitative real-time PCR (**A**) IGV diagram indicates the location of co-expressed transcripts in the same QTL_ID. (**B**) Quantitative real-time PCR validation of lncRNAs and genes in the same QTL_ID region.

Next, we tried to identify which QTL play a crucial role in fat deposition. The number of differentially expressed lncRNAs in each QTL_ID was analyzed; 65 QTL_ID containing differentially expressed lncRNAs were identified. The score for each QTL_ID were sorted (score = the number of differentially expressed lncRNAs / the span length of each QTL_ID) (Supplementary Table 9). The top 20 QTL_ID are listed in Table 4. More than half of the top 20 QTL_ID are associated with the “average backfat thickness” trait. Next, the differentially expressed mRNAs were assigned to 65 QTL_ID. This analysis indicated that gene *ELOVL6* is located in the same region of QTL_ID 21252 as lncRNA *TCONS_00185144* and *TCONS_00181156*. Moreover, the gene *STEAP4* as found in QTL_ID 21259 along with lncRNA *TCONS_00199412* and *TCONS_00197271*. Subsequently, quantitative real-time PCR confirmed their co-expression relationship (Figure 6A–6B). These results suggest that lncRNA may participate in regulations of genes in the same QTL_ID.

**Table 4:** QTL_ID ranked by number of differentially expressed lncRNA molecules

QTL_ID	DE-lncRNAs	Chrome	Trait	Name	Start	End	Score
31542	3	14	Arachidonic acid content	FA-C20:4	138365048	138414114	6.11E-05
658	1	10	Average backfat thickness	BFT	11227321	11306620	1.26E-05
12712	1	1	Abdominal fat weight	ABDF	294607712	294736101	7.79E-06
21436	1	16	Loin fat percentage	LOINFP	83843383	84125107	3.55E-06
735	1	1	Average backfat thickness	BFT	307398864	307784034	2.6E-06
17773	1	1	Average backfat thickness	BFT	140716292	141412550	1.44E-06
12713	1	1	Abdominal fat weight	ABDF	245011782	245777288	1.31E-06
22290	1	X	Average backfat thickness	BFT	113078996	113962590	1.13E-06
736	1	4	Average backfat thickness	BFT	140987596	142372300	7.22E-07
7293	3	4	Abdominal fat percentage	ABDFP	81983315	87016757	5.96E-07
22480	2	5	Arachidic acid content	FA-C20:0	56004411	59682626	5.44E-07
22509	1	10	Arachidic acid content	FA-C20:0	56004411	59682626	2.72E-07
5435	1	10	Average backfat thickness	BFT	28168636	32088890	2.55E-07
3000	2	10	Average backfat thickness	BFT	32088890	41334738	2.16E-07
7530	1	3	Average backfat thickness	BFT	122295139	126926633	2.16E-07
18001	1	9	Average backfat thickness	BFT	145703416	151394450	1.76E-07
17803	1	8	Average backfat thickness	BFT	811090	6651169	1.71E-07
23307	1	6	Backfat at rump	BFTR	152297333	158443390	1.63E-07
849	1	1	Abdominal fat weight	ABDF	226764071	233806417	1.42E-07
2923	1	13	Average backfat thickness	BFT	208227233	215641489	1.35E-07

## DISCUSSION

Fat deposition is a complex metabolic process involving many genes. Although many groups have studied genes related to backfat thickness [22–24], until now, the relationship between fat deposition and lncRNAs is not very clear. In this study, the expression of lncRNA and mRNA molecules in the adipose tissue of Luchuan and Duroc pigs were investigated and the potential regulatory role of lncRNA was analyzed.

A total of 4,868 differentially expressed lncRNA transcripts and 8843 differentially expressed mRNA transcripts were obtained from three tissues (liver, muscle, and fat). A significantly greater number of lncRNAs were found on chromosome 1 as compared to other chromosomes. In addition, the number of alternatively spliced isoforms per lncRNA molecule was significantly less than the number per mRNA molecule. These observations are in agreement with Shen et al. [25]. In addition, lncRNA molecules were of lower abundance as compared with mRNA molecules (Figure 3A). Both lncRNA and mRNA expression were strongly tissue-specific (Figure 3C–3D), which is also apparently indicated by the heat maps (Figure 4A–4B). All of results indicated that the lncRNA identified in this study have strong tissue-specific expression. Adipose tissue was found to have the greatest number of up-regulated lncRNA molecules and differentially expressed mRNA molecules. Adipose tissue data were used to perform the KEGG analysis and enrichment. A total of 794 lncRNA target genes were assigned to 226 functional signaling pathways. We focused on those target genes associated with calcium signaling.

Calcium is a key intracellular signal responsible for regulating numerous cellular processes. As an extracellular Ca^2+^ sensor, CaSR activation in the visceral white adipose tissue is associated with increase of adipose progenitor cells proliferation and elevate of adipocyte differentiation [26]. Another study reported that *Seipin* promotes fat storage in adipose tissue by regulating intracellular calcium homeostasis [27]. Both our data and that of others suggest that fat deposition is regulated by calcium signaling. Further study in this field may provide new strategies to control fat deposition.

The common method of predicting lncRNA target genes is to search a 100 kb upstream or downstream region to identify nearby protein coding regions. In order to obtain more reliable target gene information for lncRNA molecules, target genes were predicted using QTL_ID regions. We found that lncRNA *TCONS_00199412* and *TCONS_00197271* were co-expressed with gene *STEAP4* in the QTL_ID 21259 region. In addition, lncRNA *TCONS_00185144* and *TCONS_00181156* were co-expressed with *ELOVL6* (elongation of long chain fatty acids family member 6) and all three transcripts were localized to the QTL_ID 21252 region. Subsequently, their co-expression was confirmed using quantitative real-time PCR (Figure 6B). *ELOVL6* is believed to be involved in insulin resistance, lipogenesis, and obesity [28]. These results indicate the feasibility of using the QTL_ID region to predict the lncRNA target genes.

In conclusion, our data provide further basic knowledge of pig's lncRNAs. These results lay important groundwork for the further investigation of the regulatory role of lncRNA in fat deposition.

## MATERIALS AND METHODS

### Animals and samples collection

Three Luchuan and three Duroc boars were used in this study. The animals were allowed access to feed and water ad libitum and were housed under identical conditions. All pigs were sacrificed at 180 days of age, they had been performed overnight fasting before sacrificed. Three types of tissue samples (liver, muscle, and fat) were collected from three animals of each porcine breed. All animal experiments were performed under approval of the Institutional Animal Care and Use Committee (IACUC) of Guangxi University.

### RNA quantification and qualification

A total of 1.5 μg RNA per sample were obtained using TRIzol reagent (Invitrogen, USA); rRNA was removed using a Ribo-Zero rRNA Removal Kit (Epicentre, Madison, WI, USA). 1.5% agarose gels were used for monitoring RNA degradation and DNA contamination. RNA concentration and purity were analyzed using a NanoDrop 2000 Spectrophotometer (ThermoFisher Scientific, Wilmington, DE). RNA integrity was assessed using an RNA Nano 6000 Assay Kit and Agilent Bioanalyzer 2100 System (Agilent Technologies, CA, USA).

### Preparation of the lncRNA-Seq libraries

Six cDNA libraries were generated using NEBNextR UltraTM Directional RNA Library Prep Kit. First strand cDNA was synthesized using reverse transcriptase with random hexamer primers. Subsequently, DNA polymerase I and RNase H were used for second strand cDNA synthesis. The exonuclease/polymerase activities can convert overhangs into blunt ends. After adenylation was completed, ligation was performed with NEBNext Adaptor. The final 150–200 bp fragments were selected by filtration with AMPure XP Beads (Beckman Coulter, Beverly, USA). Then 3 μl USER Enzyme (NEB, USA) was used with size-selected and adaptor-ligated cDNA at 37°C for 15 min before PCR. At last, PCR products were generated using Phusion High-Fidelity DNA polymerase and purified using the AMPure XP system. An Agilent Bioanalyzer 2100 and qPCR were used to assess the quality of the libraries.

### lncRNA identification and analysis

Cufflinks software [20] was used to assemble the transcriptome. The resulting sequence was based on the reads mapped to the reference genome (Susscrofa 10.2). Then, the assembled transcripts were annotated using the Cuffcompare program (a Cufflinks package). The unknown transcripts were retained and screened for putative lncRNAs. Four computational approaches (CPC/CNCI/Pfam/cpat) were used to screen the putative lncRNAs for protein coding ability. Transcripts >200 nt and > two exons were retained as lncRNA candidates and were further screened by CPC/CNCI/Pfam/cpat that to ensure every transcript is a long non-coding RNA. At last, Cuffcompare was used to categorize the lncRNA transcripts as lincRNA, intronic lncRNA, anti-sense lncRNA and sense lncRNA.

### Quantification of expression levels and differential expression analysis

The FPKM (fragments per kilo-base of exon per million fragments mapped, calculated based on the length of the fragments and reads count mapped to the fragment) of both lncRNAs and coding genes were calculated using Cuffdiff (v2.1.1) [20] for each sample. Gene FPKMs were computed by summing the FPKMs of transcripts in each group. The *P value* was adjusted using Q value [29]. A threshold for significantly different expression was set as a Q value<0.01 and |log2(fold change)|>1.

### GO enrichment analysis and KEGG pathway enrichment analysis

Gene Ontology (GO) enrichment analysis of the differentially expressed genes (DEGs) and the target genes of differentially expressed lncRNAs was implemented using the topGO-R software packages. KOBAS [30] software was used to test the statistical enrichment of differentially expressed genes in KEGG pathways.

### Quantitative real-time PCR

Quantitative real-time PCR (qPCR) was used to validate RNA-seq results. Three replicate tissue samples from each pig were obtained and three pigs were used from each breed. Total RNA from these samples was extracted using TRIzol reagent (Invitrogen, USA); then, total RNA was purified using RNase-free DNase I (GeneStar, Beijing, China). One μg total RNA was reverse transcribed using M-MLV RNAse H-negative reverse transcriptase (Takara, Dalian, China). Finally, quantitative real-time PCR was performed on a qTOWER 3.0 real-time PCR System (Analytik-jena) with 2 × RealStar Green Fast Mixture (GeneStar, Beijing, China). The quantitative real-time PCR primer pairs used in this study are listed in Supplementary Table 10. The reaction conditions were: denaturation for 30 s at 95°C followed by 40 cycles of 95°C 15 s and 60°C 1 min. Relative gene expression levels were calculated from the Ct value and analyzed using the 2-ΔΔCT method. Samples were analyzed in triplicate to ensure their statistical significance.

## SUPPLEMENTARY MATERIALS AND TABLES





















## Abbreviations

lncRNA: long non-coding RNA
DE-lncRNAs: differentially expressed long non-coding RNAs
DE-mRNAs: differentially expressed mRNAs
QTL: quantitative trait loci
DEG: differentially expressed gene
GO: Gene Ontology
KEGG: Kyoto Encyclopedia of Genes and Genomes

## References

[1] Fukuda T, Hamaguchi M (2016). The impact of non-alcoholic fatty liver disease on incident type 2 diabetes mellitus in non-overweight individuals. Liv Int.

[2] Wang J, Zhang JG, Zheng HK, Li J, Liu DY, Li H, Samudrala R, Yu J, Wong GKS (2004). Mouse transcriptome: Neutral evolution of ‘non-coding’ complementary DNAs. Nature.

[3] Struhl K (2007). Transcriptional noise and the fidelity of initiation by RNA polymerase II. Nat Struct Mol Biol.

[4] Wilusz JE, Sunwoo H, Spector DL (2009). Long noncoding RNAs: functional surprises from the RNA world. Genes Dev.

[5] Hombach S, Kretz M (2016). Non-coding RNAs: Classification, Biology and Functioning. Adv Exp Med Biol.

[6] Flynn RL, Centore RC, O’Sullivan RJ, Rai R, Tse A, Songyang Z, Chang S, Karlseder J, Zou L (2011). TERRA and hnRNPA1 orchestrate an RPA-to-POT1 switch on telomeric single-stranded DNA. Nature.

[7] Redon S, Reichenbach P, Lingner J (2010). The non-coding RNA TERRA is a natural ligand and direct inhibitor of human telomerase. Nucleic Acids Res.

[8] Cusanelli E, Romero CA, Chartrand P (2013). Telomeric noncoding RNA TERRA is induced by telomere shortening to nucleate telomerase molecules at short telomeres. Mol Cell.

[9] Shibayama Y, Fanucchi S, Magagula L, Mhlanga MM (2014). lncRNA and gene looping: what's the connection?. Transcription.

[10] Froberg JE, Yang L, Lee JT (2013). Guided by RNAs: X-inactivation as a model for lncRNA function. J Mol Biol.

[11] Roberts TC, Morris KV, Weinberg MS (2014). Perspectives on the mechanism of transcriptional regulation by long non-coding RNAs. Epigenetics.

[12] Mourtada-Maarabouni M, Williams GT (2013). Growth arrest on inhibition of nonsense-mediated decay is mediated by noncoding RNA GAS5. Biomed Res Int.

[13] Ebralidze AK, Guibal FC, Steidl U, Zhang P, Lee S, Bartholdy B, Jorda MA, Petkova V, Rosenbauer F, Huang G, Dayaram T, Klupp J, O’Brien KB (2008). PU.1 expression is modulated by the balance of functional sense and antisense RNAs regulated by a shared cis-regulatory element. Genes Dev.

[14] Wang K, Liu F, Zhou LY, Long B, Yuan SM, Wang Y, Liu CY, Sun T, Zhang XJ, Li PF (2014). The long noncoding RNA CHRF regulates cardiac hypertrophy by targeting miR-489. Circ Res.

[15] Deng G, Sui G (2013). Noncoding RNA in oncogenesis: a new era of identifying key players. Int J Mol Sci.

[16] Li A, Zhang J, Zhou Z, Wang L, Liu Y, Liu Y (2015). ALDB: a domestic-animal long noncoding RNA database. PLoS One.

[17] Wei N, Wang Y, Xu RX, Wang GQ, Xiong Y, Yu TY, Yang GS, Pang WJ (2015). PU.1 antisense lncRNA against its mRNA translation promotes adipogenesis in porcine preadipocytes. Anim Genet.

[18] Zhao W, Mu Y, Ma L, Wang C, Tang Z, Yang S, Zhou R, Hu X, Li MH, Li K (2015). Systematic identification and characterization of long intergenic non-coding RNAs in fetal porcine skeletal muscle development. Sci Rep.

[19] Wang Y, Xue S, Liu X, Liu H, Hu T, Qiu X, Zhang J, Lei M (2016). Analyses of Long Non-Coding RNA and mRNA profiling using RNA sequencing during the pre-implantation phases in pig endometrium. Sci Rep.

[20] Trapnell C, Williams BA, Pertea G, Mortazavi A, Kwan G, van Baren MJ, Salzberg SL, Wold BJ, Pachter L (2010). Transcript assembly and quantification by RNA-Seq reveals unannotated transcripts and isoform switching during cell differentiation. Nat Biotechnol.

[21] Cantley LC (2002). The phosphoinositide 3-kinase pathway. Science.

[22] Guo X, Christensen OF, Ostersen T, Wang Y, Lund MS, Su G (2016). Genomic prediction using models with dominance and imprinting effects for backfat thickness and average daily gain in Danish Duroc pigs. Genet Sel Evol.

[23] Chen JN, Jiang YZ, Cen WM, Xing SH, Zhu L, Tang GQ, Li MZ, Jiang AA, Lou PE, Wen AX, Wang Q, He T, Zhu GX (2014). Distribution of H-FABP and ACSL4 gene polymorphisms and their associations with intramuscular fat content and backfat thickness in different pig populations. Genet Mol Res.

[24] Zang L, Wang Y, Sun B, Zhang X, Yang C, Kang L, Zhao Z, Jiang Y (2016). Identification of a 13 bp indel polymorphism in the 3’-UTR of DGAT2 gene associated with backfat thickness and lean percentage in pigs. Gene.

[25] Shen Y, Mao H, Huang M, Chen L, Chen J, Cai Z, Wang Y, Long Xu N (2016). Noncoding RNA and mRNA Expression Profiles in the Thyroid Gland of Two Phenotypically Extreme Pig Breeds Using Ribo-Zero RNA Sequencing. Genes (Basel).

[26] Bravo-Sagua R, Mattar P, Diaz X, Lavandero S, Cifuentes M (2016). Calcium Sensing Receptor as a Novel Mediator of Adipose Tissue Dysfunction: Mechanisms and Potential Clinical Implications. Front Physiol.

[27] Bi J, Wang W, Liu Z, Huang X, Jiang Q, Liu G, Wang Y, Huang X (2014). Seipin promotes adipose tissue fat storage through the ER Ca(2)(+)-ATPase SERCA. Cell Metab.

[28] Matsuzaka T, Shimano H (2009). Elovl6: a new player in fatty acid metabolism and insulin sensitivity. J Mol Med (Berl).

[29] Storey JD, Tibshirani R (2003). Statistical significance for genomewide studies. Proc Natl Acad Sci U S A.

[30] Mao X, Cai T, Olyarchuk JG, Wei L (2005). Automated genome annotation and pathway identification using the KEGG Orthology (KO) as a controlled vocabulary. Bioinformatics.

