# Supplementary material for: Comparative analyses of long non-coding RNA in lean and obese pigs
Source: Oncotarget. 2017 May 26;8(25):41440–50. doi: 10.18632/oncotarget.18269 (PMC5522191; doi:10.18632/oncotarget.18269)
Supplement: Supplementary file 1 [file oncotarget-08-41440-s001.pdf]

Comparative analyses of long non-coding RNA in lean and obese pigs

Supplementary Materials

Supplementary Table 1: All expression information of genes. See Supplementary_Table 1

Supplementary Table 2: All expression information of lncRNAs. See Supplementary_Table 2

Supplementary Table 3: The differentially expressed lncRNAs. See Supplementary_Table 3

Supplementary Table 4: The differentially expressed mRNAs. See Supplementary_Table 4

Supplementary Table 5: All of the target genes. See Supplementary_Table 5

Supplementary Table 6: The GO terms complete information. See Supplementary_Table 6

Supplementary Table 7: The KEGG pathways complete information. See Supplementary_Table 7

Supplementary Table 8: QTL location analysis of DE-lncRNAs. See Supplementary_Table 8

Supplementary Table 9: QTL-ID analysis of DE-lncRNAs and DE-mRNAs. See Supplementary_Table 9

Supplementary Table 10: The primers list

gene or lncRNA name	Forward primer(5′–3′)	Reverse primer(5′–3′)
STEAP4	GGGATTTGCCTTCCTTCACG	GACAAACACCTGGCGACTTG
ELOVL6	TCCTGGTTTCTGCTCTGTATGC	GCTCAGAGACCACAGCACTAATG
GAPDH	AGGGCATCCTGGGCTACACT	TCCACCACCCTGTTGCTGTA
TCONS_00181156	ATCATAATCACAGGAACAAATACG	AGATGTTACAGGTGTGGAAGTG
TCONS_00185144	CACTTCTGAACCAGCAGCGAG	ACGAGCAAGGAGAGGAACGTC
TCONS_00199412	GGCTTCGTTGATGAAAGTTAC	GGTTATCCCAGAGATTTAATGC
TCONS_00197271	AAAACCTCAGCCCCTTTCATC	GGGTCTTGCCCATCTCCAG
